# Research on large-scale tuberculosis screening in the elderly rural population

**DOI:** 10.1186/s12879-026-14167-9

**Published:** 2026-08-04

**Authors:** Junwei Li, Yuexia Wang, Qingrong Zhou, Ping Wang, Bing Li, Guoping Cao

**Affiliations:** 1 Department of Infectious Disease Control and Prevention, Jiangshan Center for Disease Control and Prevention, Jiangshan, Zhejiang 324100 China; 2 Department of Infectious Disease Control and Prevention, Quzhou Center for Disease Control and Prevention, Quzhou, Zhejiang 324000 China

**Keywords:** Mass screening, Tuberculosis, Diagnosis, Tuberculosis testing, Elderly

## Abstract

**Background:**

Tuberculosis (TB) continues to pose a significant public health challenge, particularly among the elderly population. To address this issue, a large-scale TB screening initiative was conducted in Jiangshan City, focusing on individuals aged 65 and older.

**Methods:**

This study conducted comprehensive screening of 72,716 elderly participants to identify TB cases. Demographic data were meticulously analyzed, and multivariate analysis was used to determine factors associated with TB. The number needed to screen (NNS) was calculated across different conditions to evaluate the cost-effectiveness of screening implementation in various risk groups.

**Results:**

During the study period, a total of 187 TB cases were diagnosed, with a prevalence of 257 cases per 100,000 population. The NNS to detect one TB case was 389 among all participants, with variations observed across different risk subgroups. Males, individuals with a BMI < 18.5, and those with a history of TB were identified as high-risk populations. Furthermore, differences in blood biochemical parameters were identified between TB patients and healthy individuals, with TB patients demonstrating slightly elevated platelet counts and lower total cholesterol levels.

**Conclusion:**

This study provides exploratory findings on the implementation of population-wide active TB screening among elderly individuals, with a non-statistically significant 10% increase in case detection compared with conventional passive surveillance systems (1.10, 95% CI: 0.85–1.43). Among male participants, the observed detection rate ratio was 1.30 (95% CI: 0.96–1.76, non-statistically significant). Our results identify three demographic and clinical parameters — male sex, poor nutritional status, and a history of previous TB — that showed cross-sectional associations with TB detection in this screening setting, suggesting they may warrant further investigation for potential inclusion in future screening protocols.

**Clinical trial:**

Not applicable.

**Supplementary Information:**

The online version contains supplementary material available at https://doi.org/10.1186/s12879-026-14167-9.

## Introduction

 Tuberculosis (TB), one of humanity’s oldest known infectious diseases, remains a persistent and substantial threat to global public health in the 21st century [1, 2]. This airborne pathogen, *Mycobacterium tuberculosis*, predominantly targets the pulmonary system while maintaining the capacity to cause disseminated disease in multiple organ systems [3–5]. Beyond its direct health consequences for infected individuals, TB represents a considerable public health hazard because of its efficient transmission dynamics within communities [6–8]. Elderly populations demonstrate particular vulnerability, with immunosenescence contributing to both increased susceptibility to primary infection and worse clinical outcomes after disease establishment [9–11]. These epidemiological realities underscore the imperative for targeted TB control measures in aging populations.

The traditional approach of diagnosing pulmonary TB only when symptomatic patients seek treatment has proven increasingly ineffective in reducing disease burden. Recent World Health Organization data show a rising global trend in TB cases [12], highlighting the urgent need for innovative control measures. Large-scale population screening has emerged as a crucial strategy [13, 14], enabling early detection and treatment of infected individuals**–**including asymptomatic cases [15]**–**through systematic testing of high-risk groups. This proactive approach not only reduces transmission risks but also minimizes long-term health impacts through timely intervention, making it particularly valuable for TB control by identifying cases before symptoms appear and improving overall cure rates.

As a representative aging city in China, Jiangshan faces growing TB control challenges due to its expanding elderly population. Physiological aging and immunocompromised conditions render older adults particularly susceptible to TB infection, with the disease typically exhibiting accelerated progression and more complicated treatment courses in this demographic [16]. To address these issues, Jiangshan has implemented a comprehensive TB screening initiative specifically targeting residents aged ≥ 65 years. This program employs early case identification and prompt intervention strategies to achieve dual objectives: (1) interrupting TB transmission chains within the community, and (2) improving health outcomes and quality of life for infected elderly individuals. Beyond its local impact, Jiangshan’s pioneering screening model offers valuable empirical evidence to inform TB control policies in other aging populations across China, thereby supporting national TB elimination efforts.

## Materials and methods

### Study population

In 2022, we launched a large-scale TB screening initiative in Jiangshan City, Zhejiang Province, China, focusing on individuals aged 65 and above. Located in western Zhejiang Province, Jiangshan City had a total population of 609,445 as of 2022, with a population density of 438 people per square kilometer. Among this population, approximately 108,375 individuals were aged 65 years or older, representing 17.78% of the total population. The city experiences a significant outflow of younger residents, leading to a relatively high proportion of elderly individuals.

### Study definitions

This study is a population-based cross-sectional tuberculosis screening study. The study employed a stratified geographical approach, categorizing participants into urban and rural administrative divisions. Body mass index (BMI) classifications followed WHO standards: underweight (< 18.5 kg/m²), normal (18.5–23.9), overweight (24.0-27.9), obese class I (28.0-29.9), and obese class II (≥ 30.0). Age stratification divided participants into three cohorts: 65–74 years, 75–84 years, and ≥ 85 years. Glycemic status was assessed using fasting plasma glucose thresholds, with levels exceeding 7.2 mmol/L defining suboptimal control [17]. Comprehensive health profiles were established through systematic documentation of tobacco and alcohol use patterns, medical histories, and biochemical analyses (including complete blood count, lipid panels, and urinalysis).

In Jiangshan City, the TB screening program for individuals aged 65 years and older has been included as one of the top ten livelihood projects of the local government. This program is conducted in conjunction with routine health check-ups for residents. Screening participants underwent chest X‑ray examination. Those whose imaging findings were suggestive of pulmonary TB were referred to a designated TB hospital for further diagnosis. All participants provided written informed consent before undergoing chest X‑ray. The X‑ray results were first independently interpreted by radiologists at the hospital where the examination was performed, while the image data were simultaneously transmitted to the imaging department of the designated hospital for a second independent reading. In cases where the two interpretations were inconsistent, a third reading was performed by a team of respiratory specialists from the designated hospital. A chest radiograph was defined as “abnormal” when the imaging findings indicated any of the following: (1) cavities and cavitary nodules; (2) exudative and proliferative lesions; (3) tuberculomas and tuberculous lesions; (4) miliary lesions; (5) pleural effusion or pleural thickening; and (6) any combination of the above abnormalities. For participants with a history of TB, their prior imaging data were retrospectively reviewed concurrently. Subsequently, patients requiring further evaluation of TB status were referred to the designated hospital, where respiratory physicians re‑performed physical examinations and conducted relevant bacteriological tests to complete the final assessment.

Case definition in this study operationalized TB diagnosis through microbiological confirmation (sputum culture positivity or acid-fast bacilli smear positivity) or physician-diagnosed cases meeting WHO clinical criteria (epidemiological linkage plus radiographic evidence of active TB) [18]. Our analysis included bacteriologically confirmed cases and clinically diagnosed cases that met the WHO diagnostic criteria. TB-related symptoms were obtained based on participant self-report, and symptom classification followed the WHO operational definition [19], with core TB-associated symptoms comprising: (1) persistent cough (> 2 weeks), (2) chest pain, (3) unintentional weight loss (> 5% of body weight in 3 months), and (4) productive sputum.

### Statistical analysis

Continuous variables were summarized as median (interquartile range, IQR), and categorical variables as frequencies and percentages. The prevalence of tuberculosis (TB) per 100,000 population and the number needed to screen (NNS) to detect one TB case were calculated for the overall population and stratified subgroups [20]. To estimate potential under-detection or healthcare access disparities, the ratio of actively screened TB prevalence to passively reported incidence was calculated by dividing the number of TB cases identified through active screening (per 100,000 population) by the number of newly notified TB cases from routine surveillance (per 100,000 population), stratified across high-risk subgroups.

For variable screening, categorical and continuous variables were compared between TB and non-TB groups using Pearson’s *χ²* test (or Fisher’s exact test) and the independent-samples t-test (or Mann–Whitney U test), respectively; variables with *P* < 0.10 were considered candidates for the multivariable model. A regularized logistic regression with L2 penalty (Ridge, C = 0.1, equivalent to λ = 10) was then applied to identify factors independently associated with TB status. All candidate variables were entered simultaneously without stepwise selection; L2 regularization also stabilized estimates for categorical variables with zero-event categories (complete separation). Categorical variables were one-hot encoded with specified reference categories, while ordinal variables were assigned consecutive numeric values and entered as continuous variables. Missing values were imputed using medians (continuous) or modes (categorical); after encoding, any residual missing values were re-imputed using column medians to ensure complete data entry.

Internal validation and statistical inference were performed using the percentile bootstrap method with 1,000 resampling iterations. In each iteration, a bootstrap sample of the same size as the training set was drawn with replacement from the training data, and the model was refitted. Based on the bootstrap coefficient distribution, adjusted odds ratios (aORs) and their 95% confidence intervals (CIs) were calculated by exponentiating the 2.5th and 97.5th percentiles. Sensitivity analyses were conducted by repeating the entire multivariable logistic regression and bootstrap procedures on two subsets: “confirmed cases + all negative controls” and “clinically diagnosed cases + all negative controls,” to assess the robustness of the findings across different TB diagnostic definitions.

## Results

A total of 72,716 participants were screened, with an approximately equal gender distribution of 34,564 males (47.53%) and 38,152 females (52.47%). Participants had a median age of 72 years (IQR 68–76), with 76.70% (*N* = 55,771) residing in township areas. Anthropometric data indicated that 62.24% (*N* = 45,262) fell within the normal weight range. Metabolic profiling revealed that 9.46% (*N* = 6,880) had diabetes, with a median fasting blood glucose level of 5.37 mmol/L (IQR 4.96-6.00). Notably, 9.51% of screened individuals (*N* = 6,916) exhibited poor glycemic control, defined as a fasting blood glucose concentrations exceeding the 7.2 mmol/L threshold.

The cohort included 14,194 active smokers (19.52%) and 17,129 current alcohol consumers (23.56%) within the cohort. Hypertension was the most prevalent comorbidity, affecting 56.56% of participants (*N* = 41,128). Clinical assessments identified 342 individuals (0.47%) presenting with TB-related symptoms, while radiographic abnormalities were detected in 23,313 participants (32.06%) during preliminary imaging evaluations. Throughout the study period, 187 TB cases were identified (Fig. 1), yielding an overall prevalence of 257 per 100,000 population, with significant gender disparity observed (139 males vs. 48 females). Comprehensive biochemical profiles from blood and urine analyses, along with additional clinical parameters, are systematically detailed in Table 1.

**Fig. 1:**
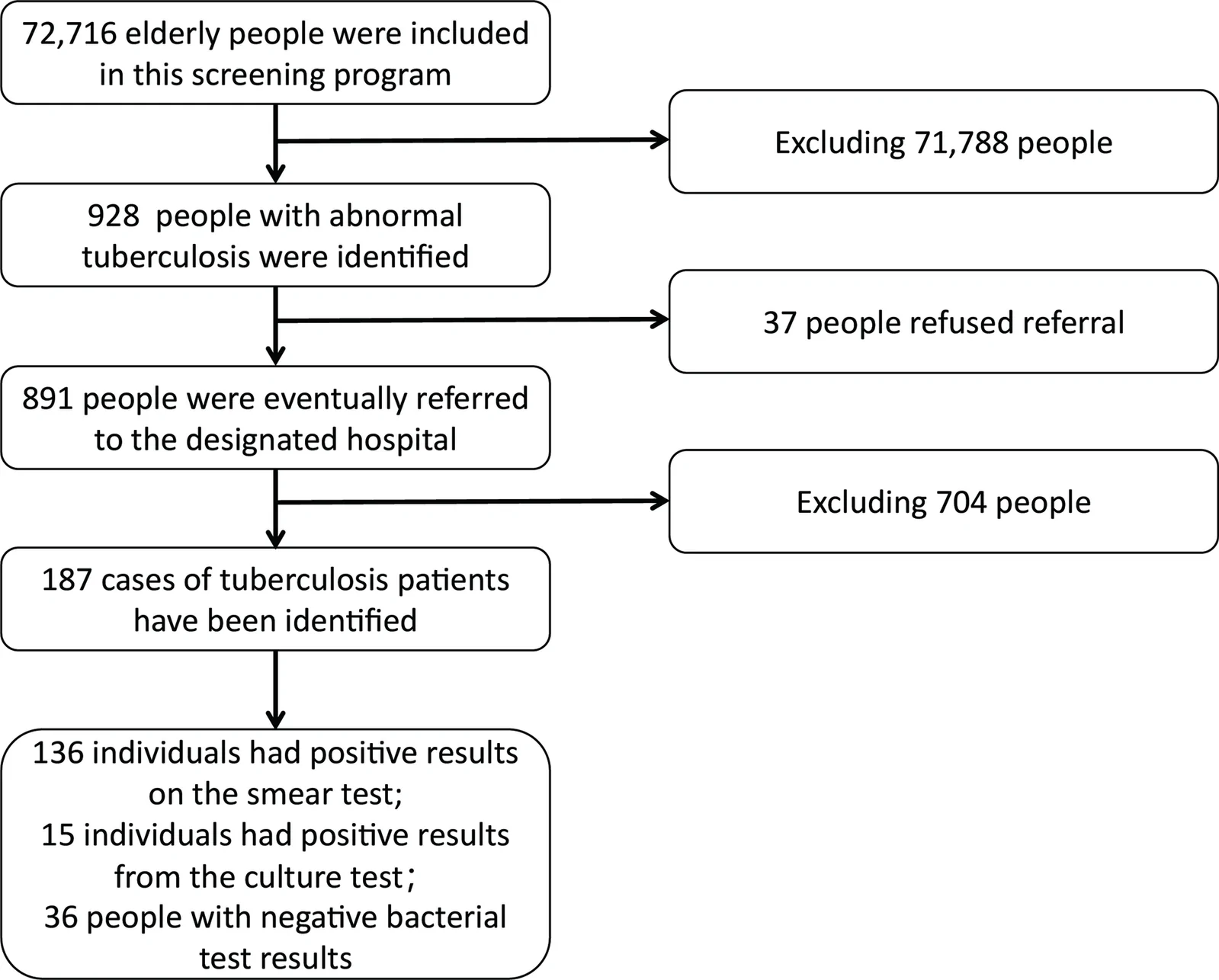
Flow chart of tuberculosis case screening

**Table 1:** Demographic characteristics and tuberculosis identification from mass tuberculosis screening of the elderly in JiangShan City

Characteristic	Male, *N* (%)	Female, *N* (%)	All participants (%)
Participant	34,564 (47.53)	38,152 (52.47)	72,716 (100)
Median age, years (IQR)	72 (68–76)	71 (68–76)	72 (68–76)
**Area**			
Urban	7772 (22.49)	9173 (24.04)	16,945 (23.30)
Town	26,792 (77.51)	28,979 (75.96)	55,771 (76.70)
**Body mass index**,** kg/m2**			
Median body mass index, kg/m2 (IQR)	22.29 (20.23–24.46)	22.64 (20.44–24.94)	22.47 (20.34–24.71)
Underweight (< 18.5)	2968 (8.59)	3212 (8.42)	6180 (8.50)
Normal weight (18.5–23.9)	22,234 (64.32)	23,028 (60.36)	45,262 (62.24)
Overweight (24.0-27.9)	7828 (22.65)	9354 (24.52)	17,182 (23.63)
Upper overweight (28.0-29.9)	1024 (2.96)	1573 (4.12)	2597 (3.57)
Obesity (≥ 30.0)	510 (1.48)	985 (2.58)	1495 (2.06)
Median fasting blood glucose mmol/L (IQR)	5.36 (4.92–6.00)	5.39 (4.99–6.01)	5.37 (4.96–6.00)
Diabetes	2549 (7.37)	4331 (11.35)	6880 (9.46)
Poor glycemic control (> 7.2 mmol/L)	3187 (9.22)	3729 (9.77)	6916 (9.51)
Cigarette (current)	14,051 (40.65)	143 (0.37)	14,194 (19.52)
Drink (current)	13,694 (39.62)	3435 (9.00)	17,129 (23.56)
Symptoms related to tuberculosis	211 (0.61)	131 (0.34)	342 (0.47)
Abnormal chest X-ray	11,742 (33.97)	11,571 (30.33)	23,313 (32.06)
hypertension	18,510 (53.55)	22,618 (59.28)	41,128 (56.56)
History of tuberculosis	312 (0.90)	121 (0.32)	433 (0.60)
**Blood biochemical indicators**			
Hemoglobin value (Hb)	140.0 (131.0–149.0)	127.0 (119.0– 134.0)	132.0 (123.0–142.0)
White blood cell count (WBC)	5.7 (4.7–6.7)	5.6 (4.7–6.6)	5.6 (4.7–6.7)
Platelet count (PLT)	173.0 (133.0–213.0)	184.0 (143.0–225.0)	179.0 (138.0–219.0)
Total cholesterol (TC)	4.67 (4.09–5.28)	5.13 (4.52–5.78)	4.91 (4.29–5.56)
Glycerin trilaurate (TG)	1.11 (0.83–1.55)	1.39 (1.03–1.93)	1.25 (0.92–1.76)
Low densith lipoprotein (LDL)	2.52 (2.03–3.05)	2.80 (2.27–3.37)	2.66 (2.14–3.22)
High density lipoprotein (HDL)	1.50 (1.24–1.82)	1.55 (1.29–1.85)	1.53 (1.26–1.83)
Total bilirubin (TBIL)	11.7 (8.8–15.6)	10.1 (7.8–13.2)	10.8 (8.2–14.4)
Glutamic-oxalacetic transaminase (AST)	26.0 (22.0–31.0)	24.0 (21.0–28.0)	25.0 (21.0–30.0)
Glutamic-pyruvic transaminase (ALT)	18.0 (13.0–24.0)	16.0 (12.0–21.0)	16.2 (13.0–22.0)
Blood creatinine levels (Cr)	77.0 (68.0–90.0)	61.0 (54.0–70.0)	69.0 (59.0–81.0)
Blood urea nitrogen (BUN)	6.16 (5.07–7.43)	5.85 (4.86–7.03)	5.99 (4.96–7.22)
Blood uric acid (BUA)	376.0 (320.0–444.0)	307.0 (258.0–364.0)	339.0 (281.0–408.0)
Alpha-fetoprotein (AFP)	2.79 (2.12–3.67)	2.58 (1.96–3.43)	2.68 (2.03–3.55)
**Urochemical indicators**			
Urine protein	10,226 (29.59)	9988 (26.18)	20,214 (27.80)
Urine sugar	1388 (4.01)	1070 (2.80)	2458 (3.38)
Urine acetone bodies	543 (1.57)	330 (0.86)	873 (1.20)
Urine occult blood	6100 (17.65)	11,076 (29.03)	17,176 (23.62)
**Confirmed cases**	139 (0.40)	48 (0.13)	187 (0.26)

### Multivariate analysis of risk factors

A total of 928 patients with high suspicion of TB were screened, of whom 891 (96.01%) were referred to designated TB hospitals. Ultimately, 187 TB patients were identified. Among them, 136 were smear-positive (72.73%), 15 (8.02%) were smear-negative but culture-positive, and 36 (19.25%) were bacteriologically negative. We first performed univariate analysis (detailed results are shown in Table S1).

A multivariable logistic regression with L2 regularization (Ridge) was fitted to identify cross-sectional associations with TB status. Internal validation and statistical inference were performed via percentile bootstrap with 1,000 resampling iterations. This model was applied to all included cases, and adjustments were made for gender, age, BMI, smoking, hypertension, tuberculosis history, and blood biomarkers. The analysis identified several factors with significant cross-sectional associations with TB status: male sex showed an adjusted odds ratio of 4.307 (95% CI: 2.607–7.785, *P* < 0.001), and a history of TB showed an aOR of 59.929 (95% CI: 28.810–142.168, *P* = 0.004). In contrast, obesity (aOR = 0.146, 95% CI: 0.044–0.412, *P* < 0.001) and BMI as a continuous variable (per 1 kg/m² increase; aOR = 0.848, 95% CI: 0.746–0.956, *P* = 0.004) were both inversely associated with TB status in this cross-sectional sample.

Hematological biomarkers showed distinct cross-sectional associations with TB status. Platelet count (aOR = 1.004, 95% CI: 1.001–1.007, *P* = 0.004) was positively associated with TB status, while total cholesterol (aOR = 0.475, 95% CI: 0.270–0.800, *P* = 0.008) and alpha‑fetoprotein (AFP; aOR = 0.966, 95% CI: 0.8469–0.9999, *P* = 0.016) were negatively associated with TB status. Detailed results are presented in Table 2.

**Table 2:** Multivariable analysis of risk factors for tuberculosis among elderly groups in JiangShan City

Characteristic	*N*, Participants (%)	All Cases			Confirmed cases			Clinical diagnosis cases		
		*N*, Number of Cases (%)	Adjusted Odds Ratio (95% CI)	*P* Value	*N*, Number of Cases (%)	Adjusted Odds Ratio (95% CI)	*P* Value	*N*, Number of Cases (%)	Adjusted Odds Ratio (95% CI)	*P* Value
Participant	72,716 (100)	187 (100.00)			151 (100.00)			36 (100.00)		
**Sex**										
Female	38,152 (52.47)	48 (25.67)	1 (Referent)		38 (25.17)	1 (Referent)		10 (27.78)	1 (Referent)	
Male	34,564 (47.53)	139 (74.33)	4.3067 (2.6071–7.7847) ^***^	< 0.001	113 (74.83)	4.4354 (2.4605–8.0706) ^***^	< 0.001	26 (72.22)	1.5987 (0.3335–8.1377)	0.512
Median age, years (IQR)	72 (68–76)	73 (69.5–77)	1.0111 (0.9768–1.0432)	0.468	72 (69.5–76)	0.9980 (0.9646–1.0312)	0.896	75 (71.25–81.75)	1.0545 (0.9752–1.1398)	0.160
**Body mass index**,** kg/m**^**2**^										
Median body mass index, kg/m2 (IQR)	22.48 (20.34–24.72)	20.03 (18.24–22.25)	0.8483 (0.7464–0.9559) ^**^	0.004	20.04 (18.35–22.28)	0.9153 (0.7873–1.0475)	0.206	19.68 (17.66–21.78)	0.6635 (0.4371–0.8720) ^***^	< 0.001
Underweight (< 18.5)	6180 (8.50)	52 (27.81)	1.5397 (0.7725–3.1895)	0.232	38 (25.17)	1.4926 (0.6435-3.3500)	0.318	14 (38.89)	1.7347 (0.3108–8.2589)	0.468
Normal weight (18.5–23.9)	45,259 (62.24)	108 (57.75)	1 (Referent)		93 (61.59)	1 (Referent)		15 (41.67)	1 (Referent)	
Overweight (24.0-27.9)	17,182 (23.63)	25 (13.37)	1.2332 (0.5356–2.7629)	0.624	19 (12.58)	1.0551 (0.4636–2.3384)	0.896	6 (16.67)	3.3614 (0.0917–40.1647)	0.256
Upper overweight (28.0-29.9)	2597 (3.57)	2 (1.07)	0.8048 (0.0194–6.5240)	0.999	1 (0.66)	0.0249 (0.0061–0.0843) ^***^	< 0.001	1 (2.78)	4.8118 (0.0599-294.4183)	0.766
Obesity (≥ 30.0) ^†^	1495 (2.06)	0	0.1456 (0.0440–0.4122) ^***^	< 0.001	0	0.0843 (0.0240–0.2407) ^***^	< 0.001	0	0.6259 (0.1793–0.9642) ^**^	0.004
Cigarette (current)	14,194 (19.52)	62 (33.16)	0.9013 (0.5452–1.4632)	0.660	52 (34.44)	0.7023 (0.3934–1.2030)	0.204	10 (27.78)	1.2276 (0.2709–4.8390)	0.758
Hypertension	41,128 (56.56)	94 (50.27)	0.9734 (0.6248–1.5183)	0.884	72 (47.68)	1.0512 (0.6574–1.7305)	0.836	22 (61.11)	0.9444 (0.3108–3.8995)	0.812
History of tuberculosis	433 (0.60)	41 (21.93)	59.9287 (28.8099–142.1680) ^**^	0.004	34 (22.52)	55.4747 (22.4606-154.0329)^***^	< 0.001	7 (19.44)	86.1602 (3.3669-1525.9993) ^*^	0.020
**Blood biochemical indicators**										
Hemoglobin value (Hb)	132.0 123.0–142.0)	129.0 (118.25–140.0)	0.9939 (0.9805–1.0074)	0.380	128.0 (118.0–139.0)	0.9814 (0.9659–0.9967) ^*^	0.016	135.0 (126.5–150.5)	1.0281 (0.9862–1.0840)	0.228
White blood cell count (WBC)	5.6 (4.7–6.7)	5.9 (4.9–7.2)	1.0969 (0.9766–1.2427)	0.114	5.9 (4.85–7.3)	1.0397 (0.8931–1.2043)	0.584	5.9 (5.15–7.0)	1.2511 (0.9262–1.6399)	0.108
Platelet count (PLT)	179.0 (138.0–219.0)	185.5 (144.25–229.75)	1.0043 (1.0012–1.0074) ^**^	0.004	185.0 (143.0–241.0)	1.0050 (1.0016–1.0085) ^**^	0.002	186.0 (151.0–205.5)	1.0010 (0.9937–1.0087)	0.752
Total cholesterol (TC)	4.91 (4.29–5.56)	4.55 (4.00–5.00)	0.4754 (0.2697–0.7994) ^**^	0.008	4.46 (3.99–4.91)	0.4461 (0.2453–0.8080) ^**^	0.006	4.80 (4.16–5.37)	0.5904 (0.1566–1.7732)	0.368
Glycerin trilaurate (TG)	1.25 (0.92–1.76)	1.12 (0.87–1.43)	1.0781 (0.7888–1.3706)	0.526	1.09 (0.86–1.34)	0.9448 (0.6593–1.2388)	0.796	1.36 (0.96–1.73)	1.1969 (0.7070–1.8185)	0.388
Low densith lipoprotein (LDL)	2.66 (2.15–3.22)	2.44 (2 0.05–2.88)	1.7063 (0.9136–3.3865)	0.088	2.43 (2.04–2.85)	1.8751 (0.9395–3.9354) (0.9207–3.6116)	0.078	2.54 (2.08–3.12)	1.3628 (0.2353–7.4768)	0.692
alpha-fetoprotein (AFP)	2.68 (2.03–3.55)	2.62 (1.96–3.57)	0.9655 (0.8469–0.9999) ^*^	0.016	2.45 (1.92–3.57)	0.9836 (0.8983-1.0000)	0.052	3.06 (2.28–3.55)	0.8113 (0.5092–0.9997) ^*^	0.016

* ***P*** < 0.05; ** ***P*** < 0.01; *** ***P*** **< 0.001.** ^†^ No cases in the obese group; estimates from regularized logistic regression with bootstrap resampling

### Number needed to screen (NNS)

The screening efficiency analysis demonstrated an overall NNS of 389 (95% CI: 333–445) to detect one TB case among all participants aged ≥ 65 years. Stratified analyses revealed improved efficiency in high-risk subgroups: males required screening of 249 individuals (95% CI: 207–290), while underweight individuals (BMI < 18.5) showed a substantially greater yield, with an NNS of 119 (95% CI: 87–151). Radiographic screening proved particularly effective, as abnormal chest imaging findings reduced the NNS to 136 (95% CI: 116–157). Most strikingly, individuals with a prior TB history exhibited exceptional screening efficiency, achieving case detection with an NNS = 11 (95% CI: 7–14). Details of the studies are summarized in Table 3.

**Table 3:** Number needed to screen to detect one tuberculosis case among elderly groups in JiangShan City

Characteristic	Risk factor	Sample	Number of screenings required to detect one case of tuberculosis (NNS)	95% confidence interval
**Participants**		72,716	389	333–445
**Risk group**				
Sex	Male	34,564	249	207–290
Body mass index	< 18.5	6183	119	87–151
Cigarette (current)	Yes	14,194	229	172–286
X-ray	Abnormal chest X-ray	23,313	136	116–157
Medical history	History of tuberculosis	433	11	7–14

### Comparison of active screening and passive diagnosis

Surveillance data from the 2022 China Statutory Infectious Disease Reporting System revealed diagnostic disparities between passive reporting and active screening approaches among elderly populations. Among 35,659 unscreened individuals aged ≥ 65 years, passive surveillance identified 83 TB cases (reporting rate = 233/100,000), with bacteriological confirmation achieved in 86.75% (*N* = 72) of cases. Comparative analysis demonstrated differences in diagnostic ascertainment: TB cases detected through active screening showed a slightly lower etiological confirmation rate (80.75% vs. 86.75%), but had a lower proportion of clinically diagnosed cases without etiological results compared with passively detected cases (0.53% vs. 6.02%).

Occupational distribution analysis demonstrated disproportionate representation among TB cases, with farmers constituting 95.72% (*N* = 179) of actively screened TB patients compared with 79.52% (*N* = 66) in passively detected cases. This demographic discrepancy primarily stemmed from enhanced passive case detection among retired individuals, who were more frequently identified through routine healthcare reporting systems rather than active screening initiatives, as detailed in Table S2. This finding highlights the influence of healthcare engagement mechanisms on TB detection across different occupational groups.

The ratio of prevalence in the active screening group to the passively diagnosed case reporting rate was (95% CI: 0.85–1.43). Among male participants, this ratio was 1.30 (95% CI: 0.96–1.76), compared with 0.91 (95% CI: 0.55–1.51) in female participants. When stratified by age, only the ≥ 85 years age group showed a ratio of 2.08 (95% CI: 0.99–4.34), although this did not reach statistical significance. Details are shown in Table 4.

**Table 4:** Comparison of prevalence and case reporting of tuberculosis in different high-risk elderly populations with active and passive detection

Target population	Number of population	Number of TB cases	Number of cases per 100,000 population	Prevalence to case notification ratio (95% CI)
**All participants**				
Active screening	72,716	187	257	1.10 (0.85–1.43)
Passive diagnosis	35,659	83	233	1 (Referent)
**Male**				
Active screening	34,564	139	402	1.30 (0.96–1.76)
Passive diagnosis	19,746	61	309	1 (Referent)
**Female**				
Active screening	38,152	48	126	0.91 (0.55–1.51)
Passive diagnosis	15,913	22	138	1 (Referent)
**Age 65–74**				
Active screening	48,850	120	246	1.32 (0.92–1.88)
Passive diagnosis	21,433	40	187	1 (Referent)
**Age 75–84**				
Active screening	19,351	50	258	0.63 (0.41–0.99)
Passive diagnosis	7611	31	407	1 (Referent)
**Age 85-**				
Active screening	4515	17	377	2.08 (0.99–4.34)
Passive diagnosis	6615	12	181	1 (Referent)

## Discussion

This investigation implemented population-based TB screening among rural residents aged ≥ 65 years-a demographic historically underrepresented in large-scale TB surveillance initiatives [21]. Active screening revealed a TB prevalence of 257/100,000, marginally exceeding the passive surveillance rate of 233/100,000, though without statistical significance. This attenuated difference may reflect the region’s elevated TB burden combined with existing healthcare infrastructure, including routine check-ups and public health campaigns that may have identified many cases through passive mechanisms. Notably, sex-stratified analysis showed that, compared with passive detection, active screening among males had a ratio of 1.30 (95% CI: 0.96–1.76), while among females the ratio was 0.91 (95% CI: 0.55–1.51). These findings suggest that, although there was no significant difference in the overall efficiency of systematic screening, targeted screening for older men may be of significant value.

This epidemiologic investigation focused on Jiangshan City-a persistent TB hotspot in Zhejiang Province, China-where systematic screening revealed a TB prevalence of 257/100,000 population. This rate substantially exceeds rates documented in lower-burden regions such as Guangdong Province (164.5/100,000 among elderly aged ≥ 65 years) [22]. The disparity between active screening yields and routine surveillance metrics underscores the insufficiency of passive case detection mechanisms, particularly in areas with entrenched TB transmission. These findings emphasize the necessity of augmenting passive reporting with systematic screening interventions to address undiagnosed infection reservoirs and mitigate persistent transmission risks in high-burden settings.

Demographic analysis of the actively screened cohort revealed pronounced gender disparities in TB burden, with males exhibiting a threefold higher prevalence than females (139 vs. 48 cases) despite a near-equal sex distribution in the study population-a finding that aligns with established epidemiological patterns of male predominance in TB morbidity [23]. Furthermore, TB-affected individuals demonstrated a marginally elevated median age compared with non-infected counterparts (73 vs. 72 years), suggesting the existence of a chronically infected reservoir among elderly residents who may have developed tolerance to subclinical presentations. This demographic, characterized by prolonged adaptation to mild symptoms and reduced health-seeking behavior, likely perpetuates community transmission through unrecognized bacillary excretion. Such subclinical infection dynamics partially explain the persistent TB transmission cycles sustaining Jiangshan City’s elevated disease burden, underscoring the critical need for enhanced case-finding strategies targeting asymptomatic carriers in high-risk populations.

Anthropometric analysis revealed metabolic differences between patients with tuberculosis and the general study population. In this cross-sectional comparison, being overweight or obese was negatively associated with tuberculosis status compared to individuals of normal weight. This pattern is consistent with the widely documented phenomena of chronic energy deficiency and metabolic wasting observed in patients with tuberculosis [23, 24].

The results of the systematic screening showed a significant association between a history of prior TB and current TB status (aOR = 59.929, 95% CI: 28.810–142.168), which is consistent with previous reports from Shanghai showing a relapse rate of 5.3% (incidence rate 7.55/1,000 person-years) among treated patients [25]. Our results support the hypothesis that individuals with prior TB history represent a higher-prevalence population that may merit prioritized screening in future targeted surveillance strategies.

Systematic biochemical analysis of this cohort study revealed marked differences in hematological parameters between TB patients and uninfected individuals. Hematological analysis showed slight elevated platelet counts and decreased total cholesterol levels in TB cases. Meanwhile, hemoglobin showed a slight decrease and low-density lipoprotein (LDL) showed an increase, though these differences were not statistically significant. These cross-sectional findings demonstrate systemic metabolic alterations associated with TB, consistent with the known inflammatory pathophysiology and chronic catabolic state of the disease, thereby providing biochemical correlates for clinical disease manifestations. The elevated leukocyte and platelet counts in TB patients are consistent with the inflammatory response to *Mycobacterium tuberculosis* infection described in the literature [26]. The infection triggers an immune response, increasing leukocyte production, and the associated inflammation may further stimulate platelet production and release [27, 28]. Concurrently, observed hypolipidemia is consistent with TB’s chronic catabolic state, where pro-inflammatory cytokines (e.g., TNF-α, IL-6) have been shown to drive adipose tissue lipolysis and intestinal malabsorption, exacerbating nutritional depletion [29, 30]. The diminished α-fetoprotein levels-a hepatic glycoprotein typically elevated during tissue regeneration-may reflect TB-associated hepatocyte dysfunction, potentially through direct mycobacterial toxicity or inflammation-induced suppression of hepatic synthetic capacity [31]. These differences in blood biochemical indices highlight the physiological changes associated with TB, particularly in the context of chronic infection and wasting. These changes may serve as useful indicators for assessing risk factors and monitoring the progression of TB disease.

Screening efficiency analysis demonstrated substantial variation in NNS across TB risk strata. Among 72,716 participants, the population-wide NNS was 389 (95% CI: 333–445), while risk-stratified subgroups showed marked improvements: males required screening 249 individuals (95% CI: 207–290), underweight subjects (BMI < 18.5) 119 (95% CI: 87–151), smokers 229 (95% CI: 172–286), and radiographically abnormal cases 136 (95% CI: 116–157). Most strikingly, individuals with a prior TB history achieved case detection with an NNS = 11 (95% CI: 7–14)-a 35-fold efficiency gain compared with general screening. These findings align with previous studies [32, 33] and establish undernutrition and TB recurrence history as paramount efficiency multipliers. The findings advocate for risk-prioritized screening frameworks that strategically target these high-yield subgroups, optimizing case-finding efficiency while conserving public health resources in TB control programs.

Our study has several limitations. First, the sample failed to cover all older adults. The observed 10% relative increase in detection yield compared with passive case notification did not reach statistical significance. Symptomatic high-risk individuals tend to seek routine clinical care rather than preventive screening, widening this gap. Enhanced community outreach and mobile screening can improve participation of hard-to-reach groups. Second, the clinic-based screening protocol introduced selection bias, systematically excluding homebound elderly individuals with limited access to healthcare facilities. This access barrier likely created an overrepresentation of healthier participants in the cohort, potentially resulting in underestimated TB prevalence. Furthermore, residual confounding is likely, as our database lacked several key covariates, including individual-level socioeconomic status, detailed history of TB exposure, and specific immunosuppressive comorbidities. Future research should strengthen data collection and analysis in these areas. Third, mass screening cost-effectiveness varies by region: it works well in high TB-burden areas but is inefficient in low-prevalence settings. Future programs should adopt spatial epidemiological data to tier screening intensity and allocate public health resources targeted at local transmission risks. Fourth, abnormal chest X-rays were defined broadly without separating active and inactive TB lesions. We could not quantify the independent diagnostic value of different radiological signs for active TB. Further studies may stratify X-ray readings, combine CT and bacteriological tests to judge lesion activity, and conduct stratified diagnostic performance analyses.

This comprehensive evaluation provides exploratory evidence on the feasibility of population-based active case-finding strategies for TB among adults aged ≥ 65 years, with a non-statistically significant 10% higher point estimate in case detection compared with passive surveillance systems. The identification of cross-sectional associations with male sex, underweight status, and prior TB history offers a potential triage framework for further investigation in high-prevalence regions. While these findings confirm the immediate diagnostic yield of systematic screening in this cohort, the translation of case detection gains into sustained epidemiological impact remains uncertain. Future implementation should prioritize longitudinal studies assessing transmission dynamics and recurrence patterns across multiple screening cycles to evaluate the intervention’s potential effects on TB transmission chains.

## Supplementary Information

Below is the link to the electronic supplementary material.

Supplementary Material 1

Supplementary Material 2

## Data Availability

The data that support the findings of this study are available on request from the corresponding author.

## Abbreviations

TB: Tuberculosis
NNS: Number needed to screen
BMI: Body mass index
IQR: Interquartile range
ORs: Odds ratios
CIs: Confidence intervals
LDL: Low-density lipoprotein
Hb: Hemoglobin value
WBC: White blood cell count
PLT: Platelet count
TC: Total cholesterol
TG: Glycerin trilaurate
HDL: High density lipoprotein
TBIL: Total bilirubin
AST: Glutamic-oxalacetic transaminase
ALT: Glutamic-pyruvic transaminase
Cr: Blood creatinine levels
BUN: Blood urea nitrogen
BUA: Blood uric acid
AFP: Alpha-fetoprotein
