# Supplementary material for: Research on large-scale tuberculosis screening in the elderly rural population
Source: BMC Infect Dis. 2026 Aug 4;26:1923. doi: 10.1186/s12879-026-14167-9 (PMC13726238; doi:10.1186/s12879-026-14167-9)
Supplement: Supplementary file 1 [file 12879_2026_14167_MOESM1_ESM.docx]

**Table S1 Comparison of Etiological Results, Diagnostic Classification, and Demographic Characteristics Between Passive Diagnosis and Active Screening in Tuberculosis Cases.**

| **Characteristic** | N, Participants (%) | N, All Cases (%) | Effect size (95% CI) ^§^ | *P* Value |
| --- | --- | --- | --- | --- |
| Participant | 72716 (100) | 187 (100.00) |  |  |
| **Sex** |  |  |  |  |
| Female | 38152 (52.47) | 48 (25.67) | 1 (Referent) |  |
| Male | 34564 (47.53) | 139 (74.33) | 3.2043 ( 2.1418 – 4.7940) ^***^ | <0.001 |
| Median age, years (IQR) | 72 (68 - 76) | 73 (69.5 - 77) | 1.2604 (0.3128 – 2.2079) ^**^ | 0.009 |
| **Area** |  |  |  |  |
| Urban | 16945 (23.30) | 37 (19.79) | 1 (Referent) |  |
| Town | 55771 (76.70) | 150 (80.21) | 1.2318 (0.8755– 1.7332) | 0.232 |
| **Body mass index, kg/m^2^** |  |  |  |  |
| Median body mass index, kg/m2 (IQR) | 22.46 (20.34 – 24.69) | 20.03 (18.24 - 22.25) | -2.1883 (-2.6452 – -1.7313) ^***^ | <0.001 |
| Underweight (<18.5) | 6180 (8.50) | 52 (27.81) | 3.5498 (2.5601 – 4.9222) ^***^ | <0.001 |
| Normal weight (18.5-23.9) | 45262 (62.24) | 108 (57.75) | 1 (Referent) |  |
| Overweight (24.0-27.9) | 17182 (23.63) | 25 (13.37) | 0.6099 (0.4197 – 0.8864) ^**^ | 0.010 |
| Upper overweight (28.0-29.9) | 2597 (3.57) | 2 (1.07) | 0.3232 (0.0205 – 5.1045) | 0.422 |
| Obesity (≥ 30.0) ^†^ | 1495 (2.06) | 0 | 0.0072 (0.0051 – 0.0101) ^***^ | <0.001 |
| Median fasting blood glucose mmol/L (IQR) | 5.37 (4.96 – 6.00) | 5.17 (4.76-6.10) | -0.0321 (-0.3243 – 0.2600) | 0.828 |
| Diabetes | 6880 (9.46) | 20 (10.70) | 1.1440 (0.7541 – 1.7354) | 0.527 |
| Poor glycemic control (> 7.2 mmol/L) | 6916 (9.51) | 22 (11.76) | 1.2662 (0.8289 – 1.9341) | 0.275 |
| Cigarette (current) | 14194 (19.52) | 62 (33.16) | 2.0468 (1.5421 – 2.7167) ^***^ | <0.001 |
| Drink (current) | 17129 (23.56) | 48 (25.67) | 1.1209 (0.8029 – 1.5651) | 0.503 |
| Symptoms related to tuberculosis | 342 (0.47) | 2 (1.07) | 2.3261 (0.1241 – 43.5885) | 0.572 |
| Hypertension | 41128 (56.56) | 94 (50.27) | 0.7760 (0.5823 – 1.034) | 0.083 |
| History of tuberculosis | 433 (0.60) | 41 (21.93) | 49.3223 (34.6714 – 70.1642) ^***^ | <0.001 |
| **Blood biochemical indicators** |  |  |  |  |
| Hemoglobin value (Hb) | 132.0 (123.0 – 142.0) | 129.0 (118.25 – 140.0) | -2.0662 (-4.4999 – 0.3675) | 0.096 |
| White blood cell count (WBC) | 5.6 (4.7 – 6.7) | 5.9 (4.9 – 7.2) | 0.5182 (0.1040 – 0.9325) ^*^ | 0.015 |
| Platelet count (PLT) | 179.0 (138.0 – 219.0) | 185.5 (144.25 – 229.75) | 14.3606 (1.7624 – 26.9588) ^*^ | 0.026 |
| Total cholesterol (TC) | 4.91 (4.29 – 5.56) | 4.55 (4.00 – 5.00) | -0.3952 (-0.5257 – -0.2646) ^***^ | <0.001 |
| Glycerin trilaurate (TG) | 1.25 (0.92 – 1.76) | 1.12 (0.87 – 1.43) | -0.2712 (-0.3557 – -0.1867) ^***^ | <0.001 |
| Low densith lipoprotein (LDL) | 2.66 (2.15 – 3.22) | 2.44 (2.05 – 2.88) | -0.2313 (-0.3410 – -0.1215) ^***^ | <0.001 |
| High density lipoprotein (HDL) | 1.53 (1.26 – 1.83) | 1.50 (1.25 – 1.75) | -0.0510 (-0.1105 – 0.0085) | 0.092 |
| Total bilirubin (TBIL) | 10.80 (8.20 – 14.40) | 10.10 (7.50 – 13.75) | -0.7024 (-1.4605 – 0.0557) | 0.069 |
| Glutamic-oxalacetic transaminase (AST) | 25.00 (21.00 – 30.00) | 25.00 (21.00 – 30.00) | -0.3155 (-1.7227 – 1.0917) | 0.659 |
| Glutamic-pyruvic transaminase (ALT) | 16.20 (13.00 – 22.00) | 16.00 (12.00 – 21.00) | -1.4249 (-3.1707 – 0.3209) | 0.109 |
| Blood creatinine levels (Cr) | 69.00 (59.00 – 81.00) | 73.00 (62.50 – 84.00) | 3.1899 (-0.6655 – 7.0452) | 0.104 |
| Blood urea nitrogen (BUN) | 5.99 (4.95 – 7.22) | 6.06 (5.07 – 7.25) | 0.1225 (-0.1957 – 0.4407) | 0.449 |
| Blood uric acid (BUA) | 339.00 (281.00 – 408.00) | 336.00 (284.00 – 410.00) | 2.9981 (-14.0442 – 20.0404) | 0.729 |
| Alpha-fetoprotein (AFP) | 2.68 (2.03 – 3.55) | 2.62 (1.96 – 3.57) | -1.0759 (-1.8920 – -0.2597) ^**^ | 0.010 |
| **Urochemical indicators** |  |  |  |  |
| Urine protein | 20214 (27.80) | 55 (29.41) | 1.0593 (0.8637 – 1.2991) | 0.580 |
| Urine sugar | 2458 (3.38) | 7 (3.74) | 1.0561 (0.8038 – 1.3875) | 0.695 |
| Urine acetone bodies | 873 (1.20) | 3 (1.60) | 1.0000 (0.2025 – 4.9382) | 0.999 |
| Urine occult blood | 17176 (23.62) | 43 (22.99) | 0.9441 (0.7807 – 1.1415) | 0.553 |

*** *P* < 0.05; ** *P* < 0.01; *** *P* < 0.001.** ^†^ No cases in the obese group, estimates from regularized logistic regression with bootstrap resampling. ^§^ Effect size refers to odds ratio (OR) for categorical variables and mean difference (MD) for continuous variables.
