# Supplementary material for: Research on large-scale tuberculosis screening in the elderly rural population
Source: BMC Infect Dis. 2026 Aug 4;26:1923. doi: 10.1186/s12879-026-14167-9 (PMC13726238; doi:10.1186/s12879-026-14167-9)
Supplement: Supplementary file 2 [file 12879_2026_14167_MOESM2_ESM.docx]

| **taxonomic category** | **Passive diagnosis (%)** | **Active screening (%)** |
| --- | --- | --- |
| **Pathogen results** |  |  |
| Tuberculosis etiology positive | 72（86.75） | 151（80.75） |
| Tuberculosis etiology negative | 6（7.23） | 35（18.72） |
| No etiological results were obtained for tuberculosis | 5（6.02） | 1（0.53） |
| **Diagnosis** |  |  |
| Clinical diagnosis cases | 11（13.25） | 36（19.25） |
| Confirmed case | 72（86.75） | 151（80.75） |
| Borderline case | 0 | 0 |
| **Crowd classification** |  |  |
| Farmer | 66（79.52） | 179（95.72） |
| Retired personnel | 12（14.46） | 4（2.14） |
| Household and unemployment | 3（3.61） | 2（1.07） |
| Commercial service | 1（1.20） | 1（0.53） |
| Worker | 1（1.20） | 1（0.53） |

**Table S2 Comparison of Etiological Results, Diagnostic Classification, and Demographic Characteristics Between Passive Diagnosis and Active Screening in Tuberculosis Cases.**
